# What's out there for parents? A systematic review of online information about prenatal microarray and exome sequencing

**DOI:** 10.1002/pd.6066

**Published:** 2021-11-07

**Authors:** Michelle Peter, Hannah McInnes‐Dean, Jane Fisher, Dagmar Tapon, Lyn S. Chitty, Melissa Hill

**Affiliations:** ^1^ NHS North Thames Genomic Laboratory Hub, Great Ormond Street Hospital for Children NHS Foundation Trust London UK; ^2^ Genetics and Genomic Medicine, UCL Great Ormond Street Institute of Child Health London UK; ^3^ Antenatal Results and Choices London UK; ^4^ Queen Charlotte's & Chelsea Hospital, Imperial College Healthcare NHS Trust London UK

## Abstract

**Objective:**

To identify what online patient information (presented in English) is available to parents about prenatal microarray (CMA) and exome sequencing (ES), and evaluate its content, quality, and readability.

**Method:**

Systematic searches (Google and Bing) were conducted, and websites were categorised according to their purpose. Websites categorised as patient information were included if they were: in English, directed at patients, or were a text, video, or online version of an information leaflet. Author‐developed content checklists, the DISCERN Genetics tool, and readability tests (the Flesch Reading Ease Score, the Gunning Fog Index, and the Simple Measure of Gobbledygook Index) were then used to assess those sources of patient information.

**Results:**

Of the 665 websites screened, 18 met the criteria. A further 8 sources were found through a targeted search of professional organisations, resulting in 26 sources available for further evaluation. In general, this was found to be low in quality, omitted details recommended by national or international guidance, and was written at a level too advanced for average readers.

**Conclusion:**

Improvements should be made to the content, quality, and readability of online information so that it both reinforces and complements the discussions between parents and clinicians about testing options during pregnancy.

## Key points

What's already known about this topic?

- The Internet is an important source of information for parents during pregnancy
- Little is known about the availability and standard of online information about newer prenatal genetic tests like chromosomal microarray (CMA) and prenatal exome sequencing (ES)

**What does this study add?**

- Our results revealed limited online information aimed at parents about CMA and ES
- The information we did identify was lacking the details recommended by professional guidelines, had low quality scores, and was written at an advanced level
- Improvements to online information for parents are needed to support informed decision‐making regarding prenatal genetic tests

## 1 INTRODUCTION

Prenatal genetic tests play an important role in identifying the underlying cause of foetal anomalies. Over the last decade, advances in genomic medicine have given rise to new technologies–such as chromosomal microarray analysis (CMA) and exome sequencing (ES), which are changing the landscape of prenatal genetic testing. With advantages over traditional diagnostic methods, including greater diagnostic yield in foetuses with multiple anomalies^1^, ^2^ and a rapid turnaround time in the delivery of results,^3^ these new techniques can have significant clinical and psychological implications for pregnancy management.

Since a definitive diagnosis following an unexpected ultrasound finding can inform genetic counselling, pregnancy management, and postnatal care, it is clear that CMA and ES offer a number of potential benefits. There are, however, limitations and unique issues that parents will need to consider when making decisions about testing. For example, there is the potential for uncertainty in the possible results arising from both CMA and ES. The volume of information extracted may increase the incidence of results that are uncertain or difficult to interpret.^4^ Parents may also receive a variant of uncertain significance (VUS) and because, in a prenatal context, phenotypic information is often incomplete, this can make interpretation and subsequent counselling a challenge.^5^ Furthermore, variants currently classified as VUS may be reclassified as pathogenic or benign as advances in technology are made.^6^ ES and CMA also have the potential to reveal findings which may have wider health implications for family members, and may disclose non‐paternity and consanguinity.^7^

Taken together, the complexities surrounding the interpretation and subsequent implications of ES and CMA results make the pre‐test counselling process very important. Clinicians need to discuss with parents all the possible types of results (taking the time to consider the patient's tolerance of uncertainty^8^), manage parents' expectations about the likelihood of receiving a genetic diagnosis, highlight any limitations with regards to clinical utility, sensitivity and specificity, all the while conveying this information in a way that can be understood. Having to absorb information at a time when they are already anxious and when there are a number of potential outcomes to weigh up (most likely with constraints on clinic time), makes it crucial that parents have access to good quality information about the test to which they can refer outside of the clinic appointment.

Increasingly, the Internet is considered an important source for pregnancy‐related information, with research indicating that the majority of pregnant women are likely to search for information online at some point during their pregnancy.^9^, ^10^, ^11^ Reasons for searching online include the desire for further knowledge,^12^ support in pregnancy decision‐making,^13^ gaining a sense of control,^14^ and seeking reassurance about pregnancy symptoms.^15^ Pertinent to the work we describe here, is that parents also cite online information‐seeking as a strategy for assuaging feelings of uncertainty surrounding the detection of a foetal anomaly^16^ and to find information about prenatal genetic testing.^17^

The standard of the pregnancy‐related information available on the Internet, however, has been called into question, with research revealing it to be highly variable,^18^ of low quality,^19^, ^20^ written at a level too advanced for the general population to understand,^21^ and lacking in pertinent details.^22^ The relative novelty of prenatal genetic tests like CMA and prenatal ES (the latter which has only recently been implemented into mainstream clinical practice), means that we know little about what is available to parents when information about prenatal genetic tests, particularly these new technologies, is sought online. Given the influential role of the Internet in supporting parental decision‐making, and the wealth of information that is discussed during pre‐test counselling, it is important that online information about prenatal genetic tests is of high quality and written at an appropriate level to allow parents to make informed decisions about and better understand their testing options.

The current study asked two questions: 1) What do parents find when they search the Internet for information about prenatal CMA and ES? 2) What is the content, quality, and readability of the patient‐directed online information for CMA and prenatal ES?

## 2 METHOD

The study is formed of two parts to answer each research question. Firstly, to identify available patient‐facing information, we conducted systematic searches using the most popular Internet search engines (Search 1) and then categorised the findings. We also performed a targeted search of websites of relevant professional bodies and organisations (Search 2). To answer research question 2, we then assessed the content, quality, and readability of the information found.

### 2.1 Design

In this study we have followed the standard process of a systematic review to identify and assess patient information by using a systematic search with defined search terms, clear inclusion and exclusion criteria applied independently by multiple researchers, and an assessment of quality^23^ (See PRISMA checklist in the Supplementary Materials). The systematic searches were conducted following the guidance for reviewing health information on the Internet laid out in Eysenbach et al.^24^ and Rew et al.^25^

### 2.2 Survey with parents to inform the search strategy

We conducted a brief survey with parents to ensure that our searches mirrored the types of searches used by parents when they look for information about invasive testing online. An invitation and the link to the online survey hosted by SurveyMonkey was posted on the online parent forum of the charity Antenatal Results and Choices (ARC) that supports parents through antenatal testing. Eighteen parents completed the survey: 94% (*n* = 17) reported searching for information about invasive testing online and 100% said they used Google for online searching. When asked what search terms they would use, 59% (*n* = 10) said they would search for ‘test in pregnancy’, 53% (*n* = 9) for ‘antenatal test’ and 24% (*n* = 4) for ‘prenatal test’. In an open‐text box, respondents added they would search for the name of the specific test offered to them, and/or the gestation at which the test was offered. Sixty‐seven per cent of respondents (*n* = 12) said they would not search for information on social media, and respondents named the website for the National Health Service (NHS; a Government‐funded healthcare service for those living in the United Kingdom) and ARC's website as trusted sources of information.

### 2.3 Ethical approval

As this study is a review of publicly available online information, ethical approval was not required. The survey with ARC parent group members was a consultation to inform the research and is not considered research, as defined by the UK Policy Framework for Health and Social Care Research. As such, it did not require review by an NHS Research Ethics Committee (http://www.hra‐decisiontools.org.uk/research/). The survey was conducted with agreement from ARC.

### 2.4 Search terms

**FIGURE 1:**
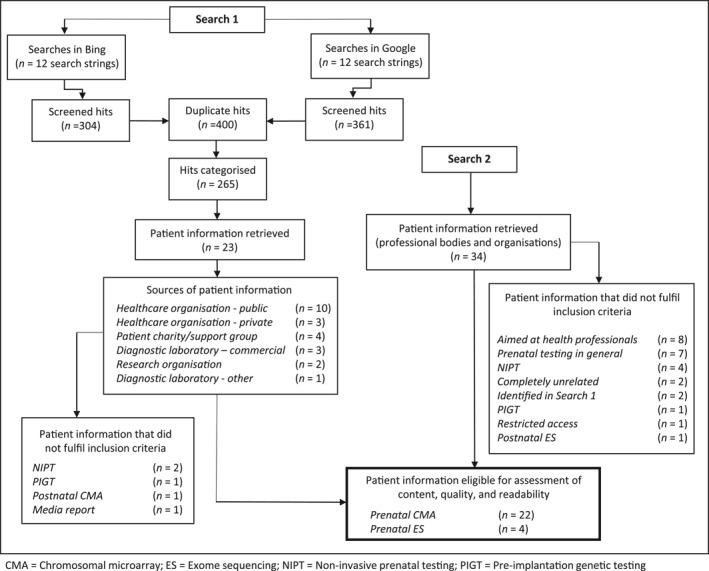
Sampling method for Searches 1 and 2

Search terms used to conduct Search 1 were informed by the experience of the research team (which includes a foetal medicine consultant, a genetic counsellor, and a patient advocate) and from responses from the parent survey. We used the following search terms: “exome sequencing”; “genome sequencing”; “microarray” and “array CGH” and paired each one with the following words: “prenatal”; “antenatal”; and “in pregnancy” to create 12 terms in total (e.g., *“prenatal exome sequencing”, “antenatal exome sequencing”, “exome sequencing in pregnancy”*). As in Skirton et al.,^22^ Boolean operators were not included between terms to reflect how parents might likely look for this information (Figure 1).

### 2.5 Sampling method

*Search 1:* Each of the 12 search terms was entered into both Google and Bing since research (both our own and by others) has shown that these are the two most popular English‐based search engines.^26^ We used depersonalised search modes in each search engine, ensuring deletion of cookies between searches. Searches were conducted on the 9th, 10th and 17th November 2020. Each researcher conducted their searches on the same day using the same computer. All searches were completed on the 17th November 2020 and no updates to these searches were performed after this time. We reviewed the first three pages from each search (since research indicates that 75% of users never scroll past the first page of search results^27^). Online information is dynamic, and so screen shots of each website eligible for analysis were saved as PDF files. Two researchers independently read through the list of findings, categorising them into broad groups according to the websites' source (e.g., journal article, healthcare organisation), purpose (e.g., academic reporting, patient information), and country of origin. Any website page that appeared more than once (whether across search engines or search terms) was classed as a duplicate hit. All duplicates were removed. Consequently, each website page was only categorised once.

*Search 2:* We manually searched the websites of relevant organisations, Government, and professional bodies relevant to prenatal genetic testing from the UK, USA, Canada, Australia and New Zealand looking for patient‐facing information on prenatal CMA and ES (see Supplementary materials). After completing Searches 1 and 2, the inclusion criteria were applied to the identified patient‐facing information.

### 2.6 Identification of patient information describing prenatal CMA and ES

Two researchers (HM, MP) independently assessed the patient information identified in Searches 1 and 2 for inclusion in an assessment of content, quality, and readability.

### 2.7 Inclusion criteria

Information was included that:

a) was directed at patients;
b) was in English;
c) described CMA or ES with a goal of supporting parental decision‐making about the test, or
d) was in the format of: i) text from a webpage, ii) a video/animation, or iii) an online version of an information leaflet.

### 2.8 Exclusion criteria

Information was excluded that:

a) was directed solely at healthcare professionals;
b) pertained only to NIPT for major trisomies, maternal serum screening, ultrasound screening, paternity testing, postnatal testing or prenatal testing in general;
c) was in the format of: i) a scientific paper, ii) a news report, or iii) a legal document;
d) was duplicated across search engines and,
e) appeared on webpages requiring registration or passwords (e.g., academic portals).

Any discrepancies between the researchers were discussed until consensus was reached.

### 2.9 Measures for assessing the content, quality, and readability of online patient information

*Content of online patient information:* We checked patient information against suggested pre‐testing counselling content from professional guidelines on CMA^28^, ^29^ and ES.^30^, ^31^, ^32^ This approach is one that has been used elsewhere in the literature.^20^, ^22^ The content guide to assess the content of patient information pertaining to CMA comprised 15 items. Each item was rated on a scale from 1 to 5 (where 1 equals “no, the information was not included, 2–4 equals “the information was partially included”, and 5 equals “yes, the information was completely included”). The total possible maximum score was 75. Patient information pertaining to ES was rated in the same way, but the content guide comprised 17 items, so the total maximum score was 85. Two researchers (HM, MP) independently rated all of the eligible patient information. A mean content score for each item and a mean total content score across all sources of information was calculated for each checklist.

*Quality of online patient information:* The DISCERN Genetics tool^33^ was used to assess the quality of the patient information. The DISCERN Genetics tool is a standardised assessment comprising 19 questions, each of which assesses a different quality criterion, plus a final question to which the user should provide a judgement on the information's overall quality. Each item is rated on a scale from 1 to 5 (where 1 equals “no, the quality criterion has not been fulfilled, 2–4 equals “the quality criterion has been partially fulfilled”, and 5 equals “yes, the quality criterion has been completely fulfilled”). Three items allow an NA rating (item not applicable) and so the total possible maximum score ranges between 85 and 100. Two researchers (HM, MP) rated all of the eligible patient information. A mean quality score for each item and a mean total quality score across all sources of information was calculated.

*Readability of online patient information:* We used three tools that have been well‐established for validating the readability of health information: The Flesch Reading Ease Score (FRES), the Gunning Fog Index (GFI), and the Simple Measure of Gobbledygook (SMOG) Index. The FRES provides an indicator of comprehension level of a piece of text. Scores range from 0 to 100; lower scores (0–30) indicate material that is complex and aimed at the level of a graduate, whilst text written at level understood by the average reader in the UK (material aimed below the reading age of a 16‐year old^34^) will score in the range of 60–70. The GFI provides an estimation of the number of years of formal education needed to understand the text in question. Shorter sentences with a basic structure receive a higher score than longer, more complicated sentences. Scores higher than 12 are considered too complex for most people. The SMOG calculates a score of readability using the number of sentences and complex words (3 or more syllables) in a piece of text. Higher scores (>14) denote complexity of the material. Scores between 7 and 12 are considered to be the reading level equivalent of 6th graders (UK school age 11–12 years), and is the level at which it has been suggested health information be aimed.^35^ Text from each source of patient information was copied into a Microsoft Word document. To maximise accuracy during analysis, any text not in a full sentence (e.g., titles, hyperlinks and bulleted points) was removed, as were references and embedded images. The remaining text from each file was then copied into an online readability programme (https://readabilityformulas.com/). Descriptive statistics for each of the readability tests and correlational analyses to examine the relationship between readability tests was calculated.

## 3 RESULTS

*Research question 1: What do parents find when they search the Internet for information about prenatal genetic tests, specifically technologies such as CMA and prenatal ES?*

Two researchers categorised the findings from Search 1 into broad themes. In line with other studies,^36^, ^37^, ^38^ interrater reliability was conducted on 10% of the data (in this case 10% = 26 website hits). Agreement between the researchers was substantial (percentage agreement = 88.5%) and greater than would be expected by chance (Cohen's kappa = 0.87, *Z* = 9.66, *p* < 0.05). Descriptive statistics provide information about the characteristics of the website pages identified (Table 1).

**TABLE 1:** Categorisation of searches (Search 1)

Categorisation of screened searches	*N* (%) hits
Websites categorised	265
**Source**	
Journal article	104 (39.2%)
Diagnostic laboratory ‐ commercial or private	32 (12.1%)
Specialist media	24 (9.1%)
Restricted access/404 error	23 (8.7%)
Healthcare organisation ‐ public sector	16 (6.0%)
Research organisation	11 (4.2%)
Patient charity or support group	10 (3.8%)
Diagnostic laboratory ‐ public sector	10 (3.8%)
Other	8 (3.0%)
General media	7 (2.6%)
Professional body/society	7 (2.6%)
Healthcare organisation – private	7 (2.6%)
Book	2 (0.8%)
Clinical trial register	2 (0.8%)
Diagnostic laboratory – other	2 (0.8%)
**Purpose**	
Academic reporting	107 (40.4%)
Resource for researchers/health professionals	54 (20.4%)
Media reporting	28 (10.6%)
Patient information	23 (8.7%)
Educational public resource	17 (6.4%)
Generic information on genetic testing	7 (2.6%)
Clinical recommendations	2 (0.8%)
Clinical trial register	2 (0.8%)
Parental support via online pregnancy forums	2 (0.8%)
Unknown	23 (8.7%)
**Country**	
USA	116 (43.8%)
UK	66 (24.9%)
China	14 (5.3%)
Israel	8 (3.0%)
Australia	6 (2.3%)
Canada	6 (2.3%)
Switzerland	4 (1.5%)
Denmark	4 (1.5%)
Cyprus	3 (1.1%)
France	3 (1.1%)
Italy	3 (1.1%)
The Netherlands	3 (1.1%)
Spain	3 (1.1%)
Korea	2 (0.8%)
Greece	1 (0.4%)
New Zealand	1 (0.4%)
Portugal	1 (0.4%)
Unknown	21 (7.9%)

Of the 23 sources of patient information that were found through Search 1, five were excluded. This was because the information: referred to NIPT (*n* = 2), pre‐implantation genetic testing (*n* = 1) or postnatal CMA (*n* = 1), or was in the form of a media report (*n* = 1). We searched the websites of 30 professional bodies and other organisations and identified eight sources of patient information. In total, both Searches 1 and 2 resulted in 26 sources of patient information being put forward for assessment of content, quality, and readability (see Supplementary materials). Of these 26, two were presented in video format and transcribed verbatim (Table 2).

**TABLE 2:** Characteristics of patient information (Searches 1 and 2) put forward for assessment

Patient information characteristics	*N* (%)
**Source**	
Healthcare organisation ‐ public sector	11 (42.3%)
Patient charity or support group	7 (26.9%)
Diagnostic laboratory ‐ commercial or private	5 (19.2%)
Healthcare organisation ‐ private sector	2 (7.7%)
Research organisation	1 (3.8%)
**Country**	
UK	15 (57.7%)
USA	6 (23.1%)
Australia	2 (7.7%)
Spain	2 (7.7%)
Israel	1 (3.8%)
**Content**	
CMA	22 (84.6%)
ES	4 (15.4%)

*Research question 2: What is the content, quality, and readability of the patient‐directed information that is available online for CMA and prenatal ES?*

*Interrater reliability analysis for content and quality of online patient information:* To provide a more robust approximation of the reliability between researchers, because the total number of patient information websites was small (*n* = 26), interrater reliability was calculated over a greater proportion of the data (25%; *n* = 7) than for the categorised findings in Search 1. The percentage agreement on each researcher's total score, Pearson's correlation between each researcher's total score, and a weighted Cohen's Kappa (quadratic) for each of the items in the assessments was calculated and indicated excellent agreement between researchers (see Supplementary materials for further details). Consequently, all scores were averaged across researchers and then used for statistical analysis.

*Analysis of the content of online patient information:* Table 3 provides information on the mean content scores by item and the total mean content score across all patient information for each checklist compared with recommended content for patient information on CMA^28^, ^29^ and ES.^30^, ^31^, ^32^ While scores across both the individual items and the total mean score showed that the overall content of patient information was lacking information recommended by professional guidelines, there was variation in the inclusion of certain topics. Most of the patient information for both CMA and ES was good at providing a simple explanation of the test, providing a timeframe for receiving results, explaining that parental samples may be needed, including information about the possibility of ‘variants of uncertain significance’ (VUS), and explaining that no result may be obtained. Very few sources, however, mentioned the possibility that CMA or ES may identify consanguinity or non‐paternity and none discussed issues of discrimination related to insurance.

**TABLE 3:** Mean content score by item for CMA and ES patient information; rated against recommended inclusions^23^, ^24^, ^25^, ^26^, ^27^

Item	CMA	Mean score (SD)	Item	ES	Mean score (SD)
1	Includes a simple explanation of the test and what it involves	3.9 (1.0)	1	Includes a simple explanation of the test and what it involves	3.8 (0.3)
2	Provides a timeframe (range) when a result can be expected	3.1 (2.0)	2	Provides a timeframe (range) when a result can be expected	3.4 (1.7)
3	Explains that CMA will not identify all genetic disorders, and includes information regarding the limitations of what can be detected by CMA	3.6 (1.1)	3	Includes information about the possibility of ‘variants of unknown significance’ (VUS) being found, and that where there is uncertainty about their significance, these variants will not be reported	1.6 (0.8)
4	Explains that CMA will identify almost all of the abnormalities that are identified by foetal karyotyping, and may identify additional specific genetic diseases	2.8 (1.4)	4	Provides realistic expectations about the chance that a clinically significant result will be obtained (e.g., gives an estimate of diagnostic yield)	2.0 (2.0)
5	Includes a discussion of possible outcomes and, where appropriate includes information on what will and will not be reported	2.7 (1.3)	5	Explains that there is a possibility that no result will be obtained (e.g., related to sample quality) and a result may not be available before the birth of the foetus in ongoing pregnancies.	3.5 (0.7)
6	Explains that diseases may be identified for which the clinical presentation may vary greatly and range from mild to severe. It may not be possible to predict what the outcome will be in a given patient.	2.2 (1.2)	6	Explains that parental samples and additional testing may be needed.	3.5 (1.9)
7	Explains that parental samples and additional testing may be needed	3.4 (1.6)	7	Discusses the inclusion or exclusion of incidental findings in the results disclosure	1.8 (1.2)
8	Includes information about the possibility of ‘variants of unknown significance’ (VUS) being found, and that where there is uncertainty about their significance, these variants will not be reported	2.5 (1.3)	8	Discusses the inclusion or exclusion of secondary findings (e.g. cancer‐susceptibility genes) in the results disclosure	1.0 (0.0)
9	Discusses the inclusion or exclusion of incidental findings in the results disclosure	1.9 (1.3)	9	Explains the handling of discoveries related to adult‐onset conditions on foetal samples	1.0 (0.0)
10	Discusses the inclusion or exclusion of secondary findings (e.g. cancer‐susceptibility genes) in the results disclosure	1.7 (1.1)	10	If incidental findings are reported, provides information on the risks of learning about incidental findings	2.0 (0.0)
11	Explains the handling of discoveries related to adult‐onset conditions on foetal samples	1.5 (0.9)	11	If incidental findings are reported, provides information about the benefits of learning about incidental findings	1.0 (0.0)
12	If incidental findings are reported, provides information on the risks of learning about incidental findings	1.1 (0.3)	12	Explains that result disclosure and post‐test counselling will be based on knowledge that is current at the time of result interpretation and disclosure	1.6 (1.3)
13	If incidental findings are reported, provides information about the benefits of learning about incidental findings	1.0 (0.0)	13	Explains that potential changes over time are likely to occur in our knowledge of disease genes, pathogenicity of sequence variants and foetal phenotypes	1.9 (1.8)
14	Explains that the test may identify consanguinity (a close blood relationship or incest) or non‐paternity	1.3 (1.0)	14	Discusses the importance of data sharing in de‐identified databases, how genetic material will be stored, and explains who will have access and for what purpose	1.3 (0.5)
15	Discusses potential issues related to insurance and discrimination	1.0 (0.0)	15	Explains that the test may identify consanguinity (a close blood relationship or incest) or non‐paternity/non‐maternity	1.0 (0.0)
			16	Explains that the results may have implications for other family members	3.1 (1.5)
			17	Discusses potential issues related to insurance and discrimination	1.0 (0.0)
					
	**Total mean score (SD)**	**32.6 (8.0)**			**32.9 (8.8)**
					

Abbreviations: CMA, chromosomal microarray analysis; ES, exome sequencing; SD, Standard Deviation.

*Analysis of the quality of online patient information:* Scores across both the individual items and the total mean DISCERN Genetics scores showed that the overall quality of patient information was low (Table 4). As with the content of patient information, however, some topics were explained better than others. For instance, the quality of the patient information was high with regards to describing the nature of the test clearly, describing the accuracy of the test results, and providing balanced and unbiased information about the test. In comparison, quality was poorer across most sources of patient information when discussing issues of discrimination related to insurance, acknowledging the psychosocial consequences of being tested, and making clear the sources of information used to develop the patient‐facing information.

**TABLE 4:** Mean DISCERN genetics score by item and total mean DISCERN score

Item	Mean score (SD)
1 Are the aims clear?	1.8 (1.2)
2 Does it achieve its aims?	3.7 (0.8)
3 Is there an explanation on the background and effects of the condition?	NA
4 Are treatment and management choices for the condition described?	NA
5 Is risk explained in simple terms?	2.0 (1.4)
6 Is the nature of the test clear?	3.7 (0.9)
7 Is the testing procedure described?	2.7 (1.1)
8 Does the information describe how accurate the test results are?	2.9 (0.8)
9 Does the information explain what happens after the test?	2.6 (1.2)
10 Does the information state who will have access to the test results?	1.8 (0.9)
11 Does the information provide support for shared decision making?	1.7 (0.8)
12 Are issues of discrimination discussed?	1.0 (0.1)
13 Does the information acknowledge the psychosocial consequences of being tested for the condition?	1.7 (1.0)
14 Are the consequences of genetic testing and screening for the relatives and partner of the person being tested discussed?	2.2 (1.0)
15 Does it provide details of additional sources of support and information?	2.2 (1.5)
16 Is it clear what sources of information were used to compile the publication?	1.3 (0.7)
17 Is it clear when the information used or reported in the publication was produced?	2.4 (1.4)
18 Is the information balanced and unbiased?	3.2 (0.9)
19 Is information provided on local availability of services and test performance?	NA
20 Based on the answers to all of the above questions, rate the overall quality of the information as a source of information about genetic testing and screening	2.5 (0.8)
**Total mean score (SD)**	**37.4 (9.2)**

*Analysis of the readability of online patient information:* The readability of online patient information was assessed using the Flesch Reading Ease Score (FRES), the Gunning Fog Index (GFI), and the Simple Measure of Gobbledygook (SMOG) Index.

All three readability tests showed that most of the patient information was aimed at a level beyond the standard of the average reader (see Table 5 and Supplementary materials). For instance, text written at the standard reading level should achieve a score of between 60 and 70 on the FRES, and lower than 12 on the GFI. In the current work, however, the mean scores were 46.1 (*SD* = 8.6) and 15.4 (*SD* = 2.0), respectively, indicating that the texts are too complex for most. Findings from our analysis using the SMOG also showed that the patient information is written at a standard beyond the 6th grade level (UK school age 11–12 years) at which it is suggested health information be aimed.^35^ These results converge with those from our subsequent correlational analyses where strong relationships between the three assessments were found (see Supplementary materials). This confirms that the online patient information identified in our searches requires patients to have literacy skills above the recommended standard.

**TABLE 5:** Descriptive statistics for the three readability tests (FRES, GFI, and SMOG)

Readability test	*N* (%)
FRES	
Difficult to read	13 (50.0%)
Fairly difficult to read	10 (38.5%)
Very difficult to read	2 (7.7%)
Standard/average	1 (3.8%)
GFI	
Hard to read	16 (61.5%)
Difficult to read	9 (34.6%)
Very difficult to read	1 (3.8%)
SMOG	
Ninth grade	1 (3.8%)
Tenth grade	7 (26.9%)
Eleventh grade	7 (26.9%)
Twelfth grade	6 (23.1%)
College	5 (19.2%)

Abbreviations: FRES, Flesch Reading Ease Score; GFI, Gunning Fog Index; SMOG, Simple Measure of Gobbledygook.

## 4 DISCUSSION

Receiving unexpected news and being offered genetic testing in pregnancy is likely to be a stressful experience for parents. Parents facing decisions in pregnancy have a great need for information and support around their options.^39^ Research has established that genetic literacy among the general public is low,^40^ and parents receiving information about prenatal genetic testing at clinical appointments may struggle to absorb everything that is said, especially if they are already distressed.^41^, ^42^ We know that patients often search online for information while trying to understand and make decisions about genetic testing and, in the United Kingdom, pregnant women have reported searching in particular for texts written by medical professionals or published by medical institutions.^43^ High‐quality written (and alternative format) information around prenatal genetic testing thus has an important role to play in facilitating informed decision‐making, understanding the test and results, and signposting options for post‐test support.

This study shows just how little is available to parents searching online for information about CMA, which has been available for many years, and the more recently‐introduced ES. The results of our searches using Google and Bing, which aimed to mirror what parents would search for, revealed that the majority of website hits were for academic articles, and only a small proportion (8.7%) were identified as relevant patient‐facing information. It is, therefore, possible that parents searching for information on prenatal genetic testing could miss useful information amongst all of the academic articles, or give up their search entirely. Signposting from clinicians on where to find relevant information is needed so that parents know where to turn should they want information to supplement what has been discussed during clinical appointments.

Like other examinations of online information relating to pregnancy,^19^, ^20^, ^21^ this study found that the quality of the information about CMA and ES was rated as low overall and had been written at an advanced reading level. We also found that, although professional bodies have made recommendations on the content that should be included when providing information to parents about CMA^28^, ^29^ and ES,^30^, ^31^, ^32^ there were several regularly occurring omissions. In particular, very few gave thorough information on the limitations and risks of CMA and ES, including the psychosocial impacts associated with testing. The initial period after learning about an unexpected finding can be a time of significant anxiety and stress.^44^ Ensuring that patient information provides details about what CMA and ES may and may not reveal can help parents manage their expectations about the likelihood of receiving a genetic diagnosis. Women undergoing CMA or ES already have uncertainty regarding the implication of the foetal abnormalities, and some who receive a VUS result have regretted undergoing CMA because of further uncertainty about the estimated risk to their child's future health.^45^ In other cases, uncertainty about results has made it difficult for parents to make decisions about whether or not to continue the pregnancy.^46^ Clinicians need to provide sufficient counselling about the range of possible outcomes (including “no findings” results which may be falsely reassuring) as well as the benefits, limitations, and wider implications of the test to promote informed decision‐making. Constraints on time, however, are a real issue: Difficulties in ensuring that such complex information is understood in the time allowed in a clinic appointment have already been encountered in paediatric care,^47^ and there are anticipated concerns about the additional time needed as ES is introduced more widely into prenatal settings.^48^ Given that couples are known to invest significant effort into researching prenatal genetic tests like CMA,^49^ and that parents undergoing prenatal ES have been reported to use the Internet as their main resource for information,^44^ it is important that online information includes sufficient content to support the discussions had during the patient‐clinician consultation. Failing to do so could mean that parents do not have a reliable additional source on which to refer once they are no longer in the presence of a clinician.

In relation to readability, it is important to note that no readability formula or tool provides a definitive measure of reading level, and that they are not tailored for medical content. The creators of the DISCERN quality criteria for information on genetic testing point out, for example, that short sentences improve readability scores but patient information is likely to have longer sentences due to the need to define genetic terms within sentences, and this will produce lower readability scores. They note, however, that embedding definitions within longer sentences is more likely to enhance the actual readability of the text by offering explanation and clarification.^33^ Nonetheless, we propose that those who develop materials about prenatal CMA and ES should consider the suggested level at which patient information should be aimed (UK school age 11–12 years^35^) since this may help parents consolidate their understanding of their testing options once their clinic appointment is over.

Most of the online patient information we found was in the format of written leaflets. However, preferences for the way in which information is presented are likely to vary. Online patient information should, therefore, be made available in different formats to take these preferences into account. Web‐based decision aids have already been used with some success to facilitate parental decision‐making about prenatal testing for chromosomal anomalies^50^, ^51^ and have also been considered an effective tool by parents considering genome sequencing for their children.^52^

There are a number of clinical and personal considerations that parents need to weigh up when making the decision to undergo prenatal genetic testing like CMA or ES. Though online patient information should be considered as an addition to, and not a substitute for, clinician counselling (particularly post testing), it remains crucial that it is written at a level that is easy for parents to understand but that does not omit important information. Furthermore, contact details for appropriate information and support services should be included.

## 5 STRENGTHS AND LIMITATIONS

A key strength of the review was the rigorous and systematic approach to identifying and reviewing patient facing‐information which was undertaken independently by two researchers with a high degree of interrater agreement. Another strength was considering the utility of the patient information from multiple perspectives: content, readability and quality. Limitations included only considering patient information available in English. There was very little information available online on ES, probably because it has only recently become available clinically. Another limitation of the study is the number of pages of search results reviewed.

## 6 CONCLUSIONS

Given parents' needs for information and support around CMA and ES, and the limitations of searching for information online, the findings indicate a need for health professionals to initiate conversations about online information and search strategies. The high number of irrelevant search results and low quality of information online are issues that should be shared with parents, and professionals should offer recommendations for appropriate websites and sources of support. As we have shown here, there is a need to develop, high quality resources to meet the needs of parents. Furthermore, parents should have input into the development of these resources which should include recommended information in a format that is understandable to a lay audience.

## CONFLICT OF INTEREST

Lyn Chitty is Editor‐in‐Chief of Prenatal Diagnosis. The remaining authors declare no conflicts of interest.

## Supporting information

Supplementary Material 1

Supplementary Material 2

## Data Availability

The data used in this study are available from the corresponding author upon request.
