# Supplementary material for: What's out there for parents? A systematic review of online information about prenatal microarray and exome sequencing
Source: Prenat Diagn. 2021 Nov 7;42(1):97–108. doi: 10.1002/pd.6066 (PMC9298227; doi:10.1002/pd.6066)
Supplement: Supplementary file 2 — Supplementary Material 2 [file PD-42-97-s001.docx]

**Table 1.** Professional bodies and organisations included in targeted search (Search 2)

| **Professional bodies and organisations** |
| --- |
| *American College of Medical Genetics and Genomics (ACMG)* |
| *American College of Obstetrics and Gynecologists (ACOG)* |
| *American Society for Reproductive Medicine (ASRM)* |
| *Antenatal Results and Choices (ARC)* |
| *Australian Genomics Health Alliance* |
| *British Society for Genetic Medicine (BSGM)* |
| *Canadian College of Medical Geneticists (CCMG)* |
| *Centre for Genetics Education - NSW Government* |
| *European Society of Human Genetics (ESHG)* |
| *Genetics Education Canada* |
| *Health Education England* |
| *Health Government New Zealand* |
| *International Society for Prenatal Diagnosis (ISPD)* |
| *International Society of Ultrasound in Obstetrics and Gynecology (ISUOG)* |
| *London North NHS Genomic Laboratory Hub* |
| *Medline Plus Genetics* |
| *Melbourne Genomics Health Alliance* |
| *National Society of Genetic Counselors (NSGC)* |
| *NHS Genomics Education* |
| *Perinatal Quality Foundation (PQF)* |
| *Royal Australian and New Zealand College of Obstetricians and Gynaecologists (RANZCOG)* |
| *Royal Australian College of General Practitioners (RACGP)* |
| *Royal College of Obstetrics and Gynaecologists (RCOG)* |
| *Royal College of Pathologists* |
| *Society for Maternal-Fetal Medicine (SMFM)* |
| *Society of Obstetricians and Gynaecologists of Canada (SOGC)* |
| *The Doctors Laboratory (TDL)* |
| *Unique* |
| *US Office on Women's Health (Dept Health & Human Services)* |
| *Victorian Clinical Genetics Services (VCGS)* |

**Table 2.** All included sources of patient information from Searches 1 and 2 (*n* = 26)

| **Website** | **Source** | **Country** | **Topic** |
| --- | --- | --- | --- |
| *Antenatal Results and Choices (ARC)* | *Patient charity or support group* | *UK* | *ES* |
| *Antenatal Results and Choices (ARC)* | *Patient charity or support group* | *UK* | *CMA* |
| *Antenatal Results and Choices (ARC)* | *Patient charity or support group* | *UK* | *ES* |
| *Antenatal Results and Choices (ARC)* | *Patient charity or support group* | *UK* | *CMA* |
| *Baylor Laboratories* | *Diagnostic laboratory - commercial or private* | *USA* | *CMA* |
| *Centre for Genetics Education* | *Healthcare organisation - public sector* | *Australia* | *CMA* |
| *Dexeus Mujer* | *Diagnostic laboratory - commercial or private* | *Spain* | *CMA* |
| *Genetic Support Foundation* | *Patient charity or support group* | *USA* | *CMA* |
| *Genetic Support Foundation* | *Patient charity or support group* | *USA* | *CMA* |
| *George Washington University* | *Research organisation* | *USA* | *CMA* |
| *Guy's and St Thomas' NHS Trust* | *Healthcare organisation - public sector* | *UK* | *CMA* |
| *Hull University Teaching Hospital NHS Trust* | *Healthcare organisation - public sector* | *UK* | *CMA* |
| *Ibbiotech* | *Diagnostic laboratory - commercial or private* | *Spain* | *CMA* |
| *Kaiser Permanente* | *Healthcare organisation - private* | *USA* | *CMA* |
| *Liverpool Women's NHS Trust* | *Healthcare organisation - public sector* | *UK* | *CMA* |
| *Mid Cheshire Hospitals NHS Trust* | *Healthcare organisation - public sector* | *UK* | *CMA* |
| *NHS North Thames Genomic Laboratory Hub* | *Healthcare organisation - public sector* | *UK* | *ES* |
| *NHS Tayside* | *Healthcare organisation - public sector* | *UK* | *CMA* |
| *Oxford NHS* | *Healthcare organisation - public sector* | *UK* | *CMA* |
| *Royal Devon and Exeter NHS Trust* | *Healthcare organisation - public sector* | *UK* | *CMA* |
| *SE of Scotland Clinical Genetic Services* | *Healthcare organisation - public sector* | *UK* | *CMA* |
| *State of Israel Ministry of Health* | *Healthcare organisation - public sector* | *Israel* | *CMA* |
| *The Doctors Laboratory (TDL)* | *Diagnostic laboratory - commercial or private* | *UK* | *CMA* |
| *UCSF Fetal Treatment Center* | *Healthcare organisation - private* | *USA* | *ES* |
| *Unique* | *Patient charity or support group* | *UK* | *CMA* |
| *Victorian Clinical Genetics Services (VCGS)* | *Diagnostic laboratory - commercial or private* | *Australia* | *CMA* |

**Table 3.** Kappa values for items on the CMA and ES checklist (assessment of interrater reliability)

| **Item** | **CMA** | **Kappa value** | **Item** | **ES** | **Kappa value** |
| --- | --- | --- | --- | --- | --- |
|  |  |  |  |  |  |
| 1 | *Includes a simple explanation of the test and what it involves* | 0.89 | 1 | *Includes a simple explanation of the test and what it involves* | NA^‡^ |
| 2 | *Provides a timeframe (range) when a result can be expected* | 0.85 | 2 | *Provides a timeframe (range) when a result can be expected* | 0.67 |
| 3 | *Explains that CMA will not identify all genetic disorders, and includes information regarding the limitations of what can be detected by CMA* | 0.83 | 3 | *Includes information about the possibility of ‘variants of unknown significance’ (VUS) being found, and that where there is uncertainty about their significance, these variants will not be reported* | 1 |
| 4 | *Explains that CMA will identify almost all of the abnormalities that are identified by fetal karyotyping, and may identify additional specific genetic diseases* | 1.00 | 4 | *Provides realistic expectations about the chance that a clinically significant result will be obtained (e.g., gives an estimate of diagnostic yield)* | 1 |
| 5 | *Includes a discussion of possible outcomes and, where appropriate includes information on what will and will not be reported* | 0.92 | 5 | *Explains that there is a possibility that no result will be obtained (e.g., related to sample quality) and a result may not be available before the birth of the fetus in ongoing pregnancies.* | NA^‡^ |
| 6 | *Explains that diseases may be identified for which the clinical presentation may vary greatly and range from mild to severe. It may not be possible to predict what the outcome will be in a given patient.* | 1.00 | 6 | *Explains that parental samples and additional testing may be needed.* | 1 |
| 7 | *Explains that parental samples and additional testing may be needed* | 0.92 | 7 | *Discusses the inclusion or exclusion of incidental findings in the results disclosure* | 0.67 |
| 8 | *Includes information about the possibility of ‘variants of unknown significance’ (VUS) being found, and that where there is uncertainty about their significance, these variants will not be reported* | 0.90 | 8 | *Discusses the inclusion or exclusion of secondary findings (e.g. cancer-susceptibility genes) in the results disclosure* | NA^‡^ |
| 9 | *Discusses the inclusion or exclusion of incidental findings in the results disclosure* | 0.89 | 9 | *Explains the handling of discoveries related to adult-onset conditions on fetal samples* | NA^‡^ |
| 10 | *Discusses the inclusion or exclusion of secondary findings (e.g. cancer-susceptibility genes) in the results disclosure* | 0.89 | 10 | *If incidental findings are reported, provides information on the risks of learning about incidental findings* | 0.67 |
| 11 | *Explains the handling of discoveries related to adult-onset conditions on fetal samples* | 1.00 | 11 | *If incidental findings are reported, provides information about the benefits of learning about incidental findings* | 1 |
| 12 | *If incidental findings are reported, provides information on the risks of learning about incidental findings* | NA^†^ | 12 | *Explains that result disclosure and post-test counselling will be based on knowledge that is current at the time of result interpretation and disclosure* | 0.5 |
| 13 | *If incidental findings are reported, provides information about the benefits of learning about incidental findings* | NA^†^ | 13 | *Explains that potential changes over time are likely to occur in our knowledge of disease genes, pathogenicity of sequence variants and fetal phenotypes* | 0.67 |
| 14 | *Explains that the test may identify consanguinity (a close blood relationship or incest) or non-paternity* | NA^‡^ | 14 | *Discusses the importance of data sharing in de-identified databases, how genetic material will be stored, and explains who will have access and for what purpose* | 1 |
| 15 | *Discusses potential issues related to insurance and discrimination* | NA^‡^ | 15 | *Explains that the test may identify consanguinity (a close blood relationship or incest) or non-paternity/non-maternity* | NA^‡^ |
|  |  |  | 16 | *Explains that the results may have implications for other family members* | 1 |
|  |  |  | 17 | *Discusses potential issues related to insurance and discrimination* | NA^‡^ |
|  | **Average kappa** | **0.92** |  | **Average kappa** | **0.83** |
|  | **(95% CI)** | **(0.88-0.95)** |  | **(95% CI)** | **(0.72-0.95)** |
| ^†^Ratings for these items included NA as a possible response. | |  |  |  |  |
| ^‡^Both researchers agreed 100% of the time which outputs an NA response. | |  |  |  |  |

**Table 4.** Kappa values for items on the DISCERN Genetics tool (assessment of interrater reliability)

| **Item** | | **Kappa value** |
| --- | --- | --- |
|  |  |  |
| 1 | *Are the aims clear?* | 0.94 |
| 2 | *Does it achieve its aims?* | 0.81 |
| 3 | *Is there an explanation on the background and effects of the condition?* | NA |
| 4 | *Are treatment and management choices for the condition described?* | NA |
| 5 | *Is risk explained in simple terms?* | 0.87 |
| 6 | *Is the nature of the test clear?* | 0.51 |
| 7 | *Is the testing procedure described?* | 0.71 |
| 8 | *Does the information describe how accurate the test results are?* | 0.67 |
| 9 | *Does the information explain what happens after the test?* | 0.84 |
| 10 | *Does the information state who will have access to the test results?* | 0.81 |
| 11 | *Does the information provide support for shared decision making?* | 0.51 |
| 12 | *Are issues of discrimination discussed?* | NA^†^ |
| 13 | *Does the information acknowledge the psychosocial consequences of being tested for the condition?* | 0.71 |
| 14 | *Are the consequences of genetic testing and screening for the relatives and partner of the person being tested discussed?* | 0.70 |
| 15 | *Does it provide details of additional sources of support and information?* | 0.74 |
| 16 | *Is it clear what sources of information were used to compile the publication?* | 1.00 |
| 17 | *Is it clear when the information used or reported in the publication was produced?* | 0.96 |
| 18 | *Is the information balanced and unbiased?* | 0.86 |
| 19 | *Is information provided on local availability of services and test performance?* | NA^†^ |
| 20 | *Based on the answers to all of the above questions, rate the overall quality of the information as a source of information about genetic testing and screening* | 0.71 |
|  |  |  |
|  |  |  |
|  | **Average kappa** | **0.77** |
|  | **(95% CI)** | **(0.66-0.89)** |
|  |  |  |
| ^†^Both researchers agreed 100% of the time which outputs an NA response. | |  |

**Table 5.** Correlations showing the relationship between readability tests

| **Readability assessment** | ***r*** | ***p*** |
| --- | --- | --- |
|  |  |  |
| *FRES and GFI* | -0.85** | 0.000 |
| *FRES and SMOG* | -0.88** | 0.000 |
| *GFI and SMOG* | 0.97** | 0.000 |
|  |  |  |
| ** Significant at *p* < .001  *r* = correlation coefficient; *p* = alpha level | | |
